# Hearing loss and intellectual outcome in children treated for embryonal brain tumors: Implications for young children treated with radiation sparing approaches

**DOI:** 10.1002/cam4.4245

**Published:** 2021-09-04

**Authors:** Iska Moxon‐Emre, Christine Dahl, Vijay Ramaswamy, Ute Bartels, Uri Tabori, Annie Huang, Sharon L. Cushing, Vicky Papaioannou, Normand Laperriere, Eric Bouffet, Donald J. Mabbott

**Affiliations:** ^1^ Program in Neuroscience and Mental Health The Hospital for Sick Children Toronto ON Canada; ^2^ Department of Psychology University of Toronto Toronto ON Canada; ^3^ Pediatric Oncology Group of Ontario Toronto ON Canada; ^4^ Division of Hematology/Oncology The Hospital for Sick Children Toronto ON Canada; ^5^ Department of Otolaryngology The Hospital for Sick Children Toronto ON Canada; ^6^ Department of Otolaryngology: Head & Neck Surgery University of Toronto Toronto ON Canada; ^7^ Department of Communication Disorders The Hospital for Sick Children Toronto ON Canada; ^8^ Radiation Oncology Ontario Cancer Institute Princess Margaret Hospital Toronto ON Canada

**Keywords:** chemotherapy, Embryonal brain tumors, intellectual outcome, pediatric cancer, sensorineural hearing loss

## Abstract

**Purpose:**

We investigate the impact of severe sensorineural hearing loss (SNHL) and for the first time evaluate the effect of unilateral versus bilateral SNHL on intellectual outcome in a cohort of children with embryonal brain tumors treated with and without radiation.

**Methods:**

Data were from 94 childhood survivors of posterior fossa (PF) embryonal brain tumors who were treated with either: (1) chemotherapy alone (*n* = 16, 7.11 [3.41] years, 11M/5F), (2) standard‐dose craniospinal irradiation (CSI) and/or large boost volumes (*n* = 44, 13.05 [3.26] years, 29M/15F), or (3) reduced‐dose CSI with a boost restricted to the tumor bed (*n* = 34, 11.07 [3.80] years, 19M/15F). We compared intellectual outcome between children who: (1) did and did not develop SNHL and (2) developed unilateral versus bilateral SNHL. A Chang grade of ≥2b that required the use of a hearing aid was considered severe SNHL. Comparisons were made overall and within each treatment group separately.

**Results:**

Patients who developed SNHL had lower full scale IQ (*p *= 0.007), verbal comprehension (*p* = 0.003), and working memory (*p *= 0.02) than patients without SNHL. No differences were observed between patients who had unilateral versus bilateral SNHL (all *p* > 0.05). Patients treated with chemotherapy alone who developed SNHL had lower mean working memory (*p = 0.03*) than patients who did not develop SNHL. Among patients treated with CSI, no IQ indices differed between those with and without SNHL (all *p *> 0.05).

**Conclusions:**

Children treated for embryonal brain tumors who develop severe SNHL have lower intellectual outcome than patients with preserved hearing: this association is especially profound in young children treated with radiation sparing approaches. We also demonstrate that intellectual outcome is similarly impaired in patients who develop unilateral versus bilateral SNHL. These findings suggest that early intervention to preserve hearing is critical.

## 1 INTRODUCTION

Curative treatment for embryonal brain tumors in children includes surgical resection, chemotherapy, and in many instances craniospinal irradiation (CSI). Unfortunately, poor neurocognitive and social outcomes are evident in survivors.^1^, ^2^, ^3^ In particular, younger age at diagnosis and higher doses of CSI and/or larger boost volumes to the brain, are associated with a decline in intellectual functioning over time.^4^, ^5^, ^6^ To reduce these adverse effects, radiation sparing treatment protocols have been developed for young children.^7^, ^8^ Although such radiation sparing approaches are associated with improved neurocognitive outcomes in young children, a sizable proportion of these vulnerable patients continue to display reduced cognitive performance.^9^

Sensorineural hearing loss (SNHL) is a common side effect of platinum‐based chemotherapy and the risk of developing SNHL increases when such chemotherapy is combined with radiation.^10^, ^11^, ^12^, ^13^ Radiation sparing protocols for children with embryonal brain tumors include repeated administration of platinum compounds. Current infant protocols employ very high doses of cisplatin, contributing to hearing loss in infants.^14^, ^15^ Due to the risk of toxicity to the ear (ototoxicity), patients undergo regular audiometric evaluations. Notably, SNHL affects quality of life as well as academic and intellectual development in all children including pediatric cancer survivors.^16^, ^17^, ^18^, ^19^, ^20^ Recent reports have identified SNHL as an important contributing factor to neurocognitive deficits in children treated with CSI for brain tumors.^21^, ^22^ For example, medulloblastoma patients treated according to a St. Jude initiated‐front‐line trial for medulloblastoma (SJMB03) who developed severe SNHL experienced significant declines in intellectual and academic outcomes compared with patients with intact hearing,^22^ and exhibited greater reading difficulties over time.^21^

Without access to sound localization via hearing, children show difficulties discriminating speech in noise, understanding speech when it is not directed toward their better hearing ear, and navigating group conversations.^23^, ^24^, ^25^ Here, we evaluate the impact of SNHL on intellectual outcome, in a cohort of patients treated for embryonal brain tumors with varying protocols (chemotherapy only, lower intensity radiation, or higher intensity radiation). Given that unilateral SNHL in children without brain tumors can lead to poor outcomes,^26^, ^27^ and that the impact of bilateral versus unilateral SNHL has not yet been examined in childhood brain tumor survivors despite being raised as a question in the literature,^13^ we also examine the impact of unilateral versus bilateral SNHL on intelligence quotient (IQ) in children treated for brain tumors. Finally, while it has been demonstrated that young children who require hearing support following treatment with radiation sparing approaches are more likely to have language deficits, impact on IQ has not been studied.^9^ By including a cohort of children treated with chemotherapy only, we test the impact of presence versus absence of SNHL on IQ in the context of radiation sparing therapy for the first time.

## 2 MATERIALS AND METHODS

### 2.1 Patients

Ninety‐four patients with embryonal posterior fossa (PF) tumors were identified for inclusion in this study. A total of 237 patients were diagnosed with embryonal brain tumors at The Hospital for Sick Children (SickKids, Toronto Canada), between 1996 and 2016. To be included in this study, patients must have survived all treatment, had a tumor located in the PF, and had neurocognitive assessment data. From the total sample of patients with embryonal tumors, we excluded 84 deceased patients and 33 patients without neurocognitive assessment data. Of the 120 children with neurocognitive assessment data, 17 were excluded for having supratentorial embryonal tumors. From the remaining sample, we only included patients with neurocognitive assessments that aligned with their audiology assessments (as detailed in the *neurocognitive assessments* section below), yielding our final sample of 94 patients. All patients from our included sample were between 5 and 17 years of age, had completed their schooling in English, had their tumors surgically resected, and were treated with protocol‐specific chemotherapy (Table S1). Patients in our final sample were grouped based on treatment protocol. The chemotherapy group (*n* = 16) consisted of patients treated with chemotherapy without radiation. We included two radiation groups, comprising of children treated with: (1) a lower radiation protocol, consisting of reduced dose CSI (23.4 Gy) and a boost restricted to the tumor bed (TB) (*n* = 34) or (2) a higher radiation protocol, consisting of standard dose CSI (30.6–39.4 Gy) and/or a boost to the entire PF (*n* = 44). This stratification was chosen in light of a previous finding that children treated with lower doses of CSI and a TB boost have a stable intellectual trajectory compared to children treated with higher doses and larger boost volumes,.^4^ Due to a protocol change at our institution, patients treated prior to 2006 received a PF boost, whereas patients treated from 2006 onward received a focal conformal boost to the TB.^4^ The demographic and medical characteristics of patients in our three treatment groups are provided in Table 1. This study was approved by the Research Ethics Board at The Hospital for Sick Children prior to commencement.

**TABLE 1:** Demographic and medical variables

	Chemotherapy		Higher radiation		Lower radiation		*p* value
	*n* = 16		*n* = 44		*n* = 34		
	*n*	(%)	*n*	(%)	*n*	(%)	
Sex							0.57
Male	11	(68.8)	29	(65.9)	19	(55.9)	
Female	5	(31.2)	15	(34.1)	15	(44.1)	
Tumor type							**<0.001**
Medulloblastoma	9	(56.2)	39	(88.6)	29	(85.3)	
ATRT	7	(43.8)	1	(2.3)	1	(2.9)	
Pineoblastoma	0	(0.0)	3	(6.8)	4	(11.8)	
PNET	0	(0.0)	1	(2.3)	0	(0.0)	
Hydrocephalus							0.995
Yes	13	(81.2)	36	(81.8)	28	(82.4)	
No	3	(18.8)	8	(18.2)	6	(17.6)	
Shunt							0.82
Yes	7	(43.8)	18	(40.9)	12	(35.3)	
No	9	(56.2)	26	(59.1)	22	(64.7)	
Sensorineural hearing loss (at time of neurocognitive testing)							**0.002**
*No SNHL*							
Chang grade <2b	6	(37.5)	15	(34.1)	25	(73.5)	
0	3	(50.0)	1	(6.7)	4	(16.0)	
1a	2	(33.3)	8	(53.3)	15	(60.0)	
1b	1	(16.7)	3	(20.2)	0	(0.0)	
2a	0	(0.0)	3	(20.0)	6	(24.0)	
*SNHL*							
Chang grade ≥2b	10	(62.5)	29	(65.9)	9	(26.5)	
2b	3	(30.0)	14	(48.3)	4	(44.4)	
3	5	(50.0)	9	(31.0)	5	(55.6)	
4	2	(20.0)	6	(20.7)	0	(0.0)	
Hearing aid							0.06
Yes	8	(50.0)	12	(27.3)	6	(17.6)	
No	8	(50.0)	32	(72.7)	28	(82.4)	
	Mean	(SD)	Mean	(SD)	Mean	(SD)	
Chemotherapy							
∑CDDP, mg/m^2^	271.61^a^	(104.36)	347.40^a,b^	(121.35)	273.97^b^	(71.44)	**0.003**
∑Carboplatin g/m^2^	2081.94^c,d^	(1050.74)	58.64^c^	(276.70)	159.12^d^	(621.84)	**<0.001**
Age at testing, years	7.11^e,f^	(3.41)	13.05^e,g^	(3.26)	11.07^f,g^	(3.80)	**<0.001**
*No‐SNHL*	6.37	(3.78)	12.85	(2.81)	11.31	(3.97)	
*SNHL*	7.56	(3.28)	13.15	(3.52)	10.40	(3.50)	
Age at diagnosis, years	2.61^h,i^	(1.05)	7.76^h^	(3.63)	7.61^i^	(3.80)	**<0.001**
*No‐SNHL*	2.67	(0.26)	8.20	(3.40)	8.15	(3.80)	
*SNHL*	2.57	(1.33)	7.53	(3.78)	6.10	(3.60)	
Time since diagnosis, years	4.52	(3.60)	5.29^j^	(3.28)	3.60^j^	(1.66)	**0.04**
*No‐SNHL*	3.72	(3.83)	4.65	(2.45)	3.17	(1.49)	
*SNHL*	4.99	(3.58)	5.62	(3.64)	4.80	(1.59)	
Time since SNHL, years	3.57	(2.89)	2.84	(2.78)	3.31	(1.27)	0.72

Notes

ATRT, atypical teratoid rhabdoid tumor; CDDP, cisplatin; SNHL, sensorineural hearing loss (Chang grade <2b = No‐SNHL; Chang grade ≥2b = SNHL), Chang grade breakdowns (0–4) are based on ratings from patients’ most impaired ear; PNET, primitive neuroectodermal tumor; SD, standard deviation. Matching letters indicate groups that differed in the three group comparisons: ^a^ *p* = 0.04, ^b^ *p* = 0.007, ^c^ *p* < 0.001, ^d^ *p* < 0.001, ^e^ *p* < 0.001, ^f^ *p* = 0.001, ^g^ *p* = 0.05, ^h^ *p* < 0.001, ^i^ *p* < 0.001, ^j^ *p* = 0.03.

Bolded values indicate significant *P*‐values (i.e. *P* < 0.05)

### 2.2 Audiology assessments

**FIGURE 1:**
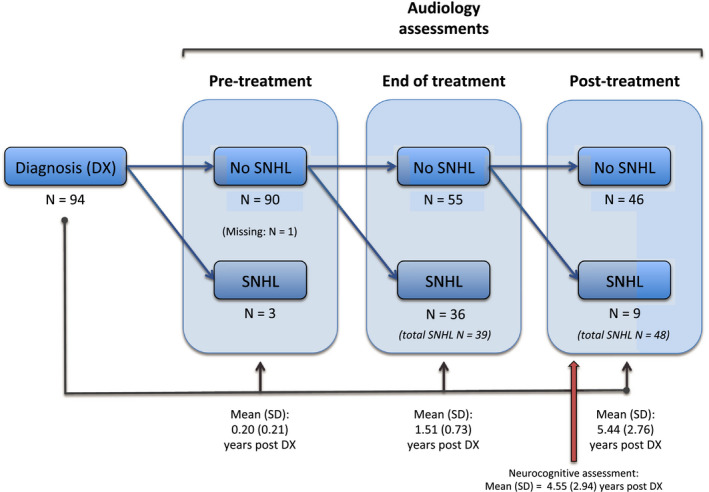
Hearing status of patients at each audiology assessment. Pre‐treatment, prior to commencement of chemotherapy, but following surgery and radiotherapy; end of treatment, immediately following completion of all therapy; post‐treatment, last documented audiology assessment where hearing was still intact, or first audiology assessment following the end of treatment assessment where SNHL was detected. Each audiology assessment box characterizes hearing status of patients from the previous assessment point that did not have evidence of SNHL (e.g., ‘post‐treatment’ assessment box characterizes the 55 patients who did not have SNHL at ‘end of treatment’, whereas the 36 participants with SNHL at ‘end of treatment’ were not further characterized because SNHL was already detected). Patients were classified as having SNHL based on the first assessment point where SNHL was detected, and change over time beyond this point was not captured. Three patients had SNHL at their pre‐treatment assessment, as a result of the tumor itself

Patients had regular audiology assessments as per their treatment protocols. Across these assessments, we abstracted hearing status at three time points for each patient: (1) pre‐treatment: prior to commencement of chemotherapy, but following surgery and CSI (mean time since diagnosis = 0.20 [0.21] years), (2) immediately following completion of all therapy (mean time since diagnosis = 1.51 [0.73] years), and (3) post‐treatment: corresponding to either the last documented assessment where hearing was still intact, or the first assessment where SNHL was detected (mean time since diagnosis = 5.44 [2.76] years). The rationale for recording the latest assessment documenting intact hearing was to ensure children in the No‐SNHL group were SNHL‐free at the time of neurocognitive assessment. In contrast, we recorded the first assessment where SNHL was detected, to characterize how long children in the SNHL group have been living with hearing loss at the time of neurocognitive assessment. The breakdown of patients with and without SNHL at each audiology assessment is detailed in Figure 1.

Hearing assessments were performed using pure tone audiometry, and audiograms were evaluated blinded to neurocognitive test results. Data from audiometry tests were graded according to the Chang Ototoxicity scale.^28^ A Chang grade of ≥2b that required the use of a hearing aid was considered severe SNHL. Thus, the Chang grade of 2b was used as a threshold for categorizing patients as having severe SNHL or not.

### 2.3 Neurocognitive assessment

For each patient, data from a single intellectual assessment were used (mean time since diagnosis = 4.55 [2.94] years). Data were extracted from the latest neurocognitive assessment available for each patient in order to capture the long‐term effects of SNHL on intellectual outcome. The only exceptions were: (1) if the assessment did not contain any measures of intellectual functioning, in which case an earlier assessment was used or (2) for the small subset of patients where SNHL was first documented in the longer term (on average >5 years post diagnosis; Figure 1), only assessments that were conducted >12 months prior to developing SNHL were used. Although the latest available assessments for this subsample were occasionally close in time to when SNHL was recorded for the first time, we included these patients in our SNHL group on the basis that SNHL likely developed before it was recorded clinically (audiology follow‐up became less frequent in the longer term).

Measures to assess intellectual function included the Wechsler Intelligence Scale for Children (WISC; editions III, IV, and V), Wechsler Preschool and Primary Scale of Intelligence (WPPSI; editions III and IV), Wechsler Abbreviated Scale of Intelligence (WASI), Wechsler Adult Intelligence Scale (WAIS; edition IV), and the Woodcock Johnson Tests of Cognitive Abilities (WJ; edition III).^29^ Patients younger than 6 years of age were administered the WPPSI, whereas patients treated on the SJBM03 protocol were routinely administered the WJ. However, patients treated on the SJMB03 protocol often had routine neurocognitive assessments containing the WISC or WASI, and when available, these were used instead. Multiple test versions were included as these changed over the time frame captured in this study. The measures administered to patients in each treatment group, and according to SNHL status, are detailed in Table S2. Overall, intellectual ability was assessed using: Full‐Scale IQ score (FSIQ); Verbal Comprehension Index measuring verbal reasoning abilities; Perceptual Reasoning Index assessing the ability to interpret and organize non‐verbal information; Working Memory Index assessing the ability to remember new information as well as concentration abilities; and the Processing Speed Index evaluating grapho‐motor and mental processing speed.

Children who had been prescribed hearing aids were asked to wear their hearing aids throughout the neurocognitive assessment. Assessments were conducted 1:1 in a quiet room and the examiner frequently checked with the child to ensure that auditory stimuli was of sufficient volume. Audiology assessments were reviewed by the clinical neuropsychologist prior to the neurocognitive assessment, and it was deemed reasonable to assess intelligence using the age appropriate scales.

### 2.4 Statistical analyses

#### 2.4.1 Medical and demographic variables

Chi‐squared analyses were performed to examine treatment group differences in sex, diagnosis, and the number of patients who developed hydrocephalus, required a shunt, developed SNHL, or wore a hearing aid. Univariate analyses of variance (ANOVAs) were performed to evaluate if the three treatment groups differed in their age at diagnosis, time since diagnosis, time since developing SNHL, cumulative cisplatin dose, or cumulative carboplatin dose.

#### 2.4.2 Intellectual outcome in the full sample

A series of univariate analyses of covariance (ANCOVAs) were conducted to evaluate the effect of: (1) hearing status (SNHL and no‐SNHL) and (2) treatment (chemotherapy, higher radiation, and lower radiation), on multiple indices of intellectual outcome (full scale IQ, verbal comprehension, perceptual reasoning, working memory, and processing speed) within the entire patient sample (*n* = 94). Age at diagnosis and time since diagnosis were included as covariates because they differed between the treatment groups (Table 1).

#### 2.4.3 Intellectual outcome in patients with SNHL

A separate series of ANCOVAs were conducted in the subsample of patients who developed SNHL (*n* = 48), to evaluate the impact of: (1) laterality (unilateral and bilateral SNHL) and (2) treatment (chemotherapy, lower radiation, and higher radiation), on multiple indices of intellectual outcome. Age at diagnosis was included as a covariate because it differed between the treatment groups in this cohort (Table S3).

#### 2.4.4 Exploratory analyses—comparing intellectual outcome between patients with SNHL versus no‐SNHL, within each treatment group

Due to the small sample sizes among the treatment groups stratified by hearing status (SNHL and no‐SNHL), a series of Mann–Whitney *U* tests were conducted to compare the impact of hearing status on each measure of intellectual outcome, within each treatment group separately. No covariates were included.

For all ANCOVAs, post hoc pairwise comparisons were corrected for multiple comparisons, using the modified Hochberg procedure. For Mann‐Whitney U tests, individual p‐values were corrected using the modified Hochberg procedure, to account for the multiple comparisons made.

## 3 RESULTS

### 3.1 Patient demographic/medical variables and hearing status

Patient demographic and medical variables are provided in Table 1. The three groups (chemotherapy, higher radiation, and lower radiation) did not differ in sex (*p *= 0.57), time since SNHL (*p* = 0.72), presence of hydrocephalus (*p *= 0.995), or requiring a shunt (*p* = 0.82). While a greater proportion of patients treated with chemotherapy only used hearing aids, this was not significant (*p *= 0.06). Tumor type was different across groups (*p* > 0.001); namely, the incidence of atypical teratoid rhabdoid tumor (ATRT) was greatest, and the incidence of medulloblastoma was lowest, in the chemotherapy only group. Consistent with the current treatment approach, patients in the chemotherapy group were younger at time of diagnosis, and younger at time of neurocognitive testing, compared to patients in either radiation group (all *p *< 0.001). Notably, hearing status differed across groups (*p* = 0.002); with the lower radiation group having the smallest proportion of patients who developed SNHL.

### 3.2 Intellectual outcome in the full sample

#### 3.2.1 Patients who develop SNHL have lower intellectual functioning overall

**FIGURE 2:**
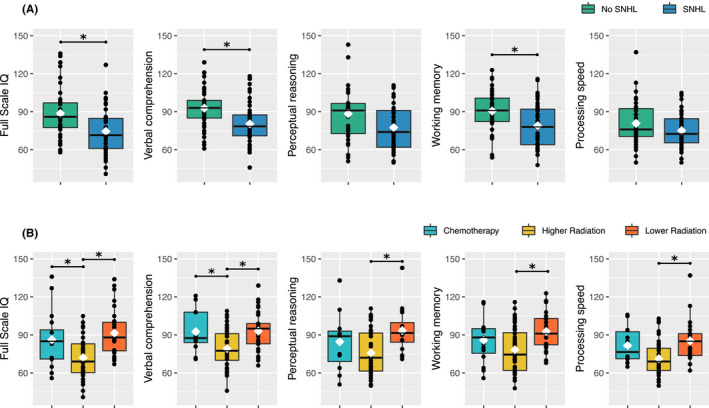
Boxplots showing all data points for measures of intellectual function in: (A) patients stratified by hearing status (SNHL or No SNHL). Irrespective of treatment, patients with SNHL have lower scores on all measures of intellectual functioning (except for processing speed), than patients without SNHL; (B) patients stratified by treatment (chemotherapy, higher radiation or lower radiation). Irrespective of hearing status, patients treated on higher radiation protocols have the poorest intellectual outcomes. Black line, median; white diamond, mean, *p < 0.05. Refer to Table 2 for complete model results. *Note*: means plotted here are unadjusted, whereas the group means presented in Table 2 account for age at diagnosis and time since diagnosis

When all patients were considered together, those who developed SNHL had lower full scale IQ (*p *= 0.007), verbal comprehension (*p* = 0.003), and working memory (*p* = 0.02); (Table 2, Figure 2A) than patients without SNHL. In contrast, perceptual reasoning (*p *= 0.11) and processing speed (*p* = 0.30) did not differ between patients who did and did not develop SNHL (Table 2, Figure 2A).

**TABLE 2:** Intellectual outcome in the full sample

Full scale IQ										*F* value	*p* value
Hearing status	No‐SNHL (<2b)			SNHL (≥2b)							
	Mean	SE	*n*	Mean	SE	*n*					
	86.66	2.64	43	76.38	2.55	46				7.89	**0.007**
Treatment	Chemotherapy			Higher radiation			Lower radiation				
	Mean	SE	*n*	Mean	SE	*n*	Mean	SE	*n*		
	88.95^a^	4.76	16	73.28^a,b^	2.69	42	88.35^b^	3.02	31	7.81	**<0.001**
Verbal comprehension										*F* value	*p* value
Hearing status	No‐SNHL (<2b)			SNHL (≥2b)							
	Mean	SE	*n*	Mean	SE	*n*					
	91.49	2.26	45	81.71	2.18	48				3.05	**0.003**
Treatment	Chemotherapy			Higher radiation			Lower radiation				
	Mean	SE	*n*	Mean	SE	*n*	Mean	SE	*n*		
	97.12^c^	4.10	16	78.90^c,d^	2.28	44	91.32^d^	2.53	33	9.47	**<0.001**
Perceptual reasoning										*F* value	*p* value
Hearing status	No‐SNHL (<2b)			SNHL (≥2b)							
	Mean	SE	*n*	Mean	SE	*n*					
	86.38	3.40	30	78.99	2.89	41				2.66	0.11
Treatment	Chemotherapy			Higher radiation			Lower radiation				
	Mean	SE	*n*	Mean	SE	*n*	Mean	SE	*n*		
	89.93	6.18	11	75.83^e^	2.95	40	90.38^e^	4.05	20	4.61	**0.01**
Working memory										*F* value	*p* value
Hearing status	No‐SNHL (<2b)			SNHL (≥2b)							
	Mean	SE	*n*	Mean	SE	*n*					
	88.95	2.56	42	80.36	2.42	47				5.74	**0.02**
Treatment	Chemotherapy			Higher radiation			Lower radiation				
	Mean	SE	*n*	Mean	SE	*n*	Mean	SE	*n*		
	89.09	4.77	15	78.67^f^	2.52	44	90.51^f^	2.99	30	4.98	**0.009**
Processing speed										*F* value	*p* value
Hearing status	No‐SNHL (<2b)			SNHL (≥2b)							
	Mean	SE	*n*	Mean	SE	*n*					
	79.59	2.43	38	76.14	2.21	46				1.08	0.30
Treatment	Chemotherapy			Higher radiation			Lower radiation				
	Mean	SE	*n*	Mean	SE	*n*	Mean	SE	*n*		
	83.07	4.25	14	71.87^g^	2.23	42	83.77^g^	2.68	28	6.51	**0.002**

Note

Results from ANCOVAs, comparing intellectual outcome across patients (*n* = 94) stratified by: (1) hearing status (sensorineural hearing loss [SNHL]/no‐SNHL; Chang grade ≥2b = severe SNHL) and (2) treatment (chemotherapy/higher radiation/lower radiation). Age at diagnosis and time since diagnosis were included as covariates, thus adjusted group means and standard errors (SE) are provided. Due to differences in tests used to assess intellectual outcome, *n*'s vary across groupings. Post hoc pairwise analyses were corrected for multiple comparisons using the modified Hochberg procedure. Matching letters indicate significant post hoc pairwise comparisons among groups from the three group comparison: ^a^ *p* = 0.02, ^b^ *p* = 0.001, ^c^ *p* = 0.001, ^d^ *p* = 0.01, ^e^ *p* = 0.02, ^f^ *p* = 0.01, ^g^ *p* = 0.003. n = sample size.

#### 3.2.2 Patients in the chemotherapy and lower radiation groups have more favorable intellectual outcomes than patients in the higher radiation group

There was a significant main effect of treatment group, across multiple indices of intellectual outcome (all *p *< 0.05; Table 2). Post hoc analyses revealed that patients treated with chemotherapy alone had higher full‐scale IQ (*p *= 0.02) and verbal comprehension (*p* = 0.01) scores than patients treated with higher radiation protocols (all *p *< 0.05; Table 2, Figure 2B). Patients treated on lower radiation protocols had higher scores across multiple indices of intellectual outcome (all *p *< 0.05) than patients treated on higher intensity protocols (Table 2, Figure 2B). The chemotherapy and lower radiation groups did not differ on any measure of intellectual outcome (Table 2).

### 3.3 Intellectual outcome in patients with SNHL only

#### 3.3.1 Patients with unilateral and bilateral SNHL have comparable intellectual outcomes

None of the measures of intellectual outcome differed between patients who had unilateral versus bilateral SNHL, in the overall subsample (all *p *> 0.05).

#### 3.3.2 In the subsample of patients with SNHL, the chemotherapy and lower radiation groups have more favorable outcomes than the higher radiation group

Patients in the chemotherapy group had higher full scale IQ (*p *= 0.05) and verbal comprehension (*p* = 0.004) scores than patients in the higher radiation group (Table 3). Patients in the lower radiation group had higher verbal comprehension (*p* = 0.02) and processing speed (*p* = 0.05) scores than the higher radiation group (Table 3). Perceptual reasoning and working memory did not differ between the treatment groups (all *p* > 0.05; Table 3).

**TABLE 3:** Intellectual outcome in subset of patients with SNHL

Full scale IQ										*F* value	*p* value
Laterality	Unilateral SNHL			Bilateral SNHL							
	Mean	SE	*n*	Mean	SE	*n*					
	74.38	5.36	11	74.57	3.00	35				0.001	0.98
Treatment	Chemotherapy			Higher radiation			Lower radiation				
	Mean	SE	*n*	Mean	SE	*n*	Mean	SE	*n*		
	85.89^a^	5.79	10	68.28^a^	3.17	29	84.13	6.14	7	4.56	**0.02**
Verbal comprehension										*F* value	*p* value
Laterality	Unilateral SNHL			Bilateral SNHL							
	Mean	SE	*n*	Mean	SE	*n*					
	78.60	4.68	11	81.01	2.55	37				0.20	0.65
Treatment	Chemotherapy			Higher radiation			Lower radiation				
	Mean	SE	*n*	Mean	SE	*n*	Mean	SE	*n*		
	96.36^b^	4.61	10	73.06^b,c^	2.53	29	86.63^c^	4.34	9	10.01	**<0.001**
Perceptual reasoning										*F* value	*p* value
Laterality	Unilateral SNHL			Bilateral SNHL							
	Mean	SE	*n*	Mean	SE	*n*					
	74.27	5.72	10	78.53	3.23	31				0.42	0.52
Treatment	Chemotherapy			Higher radiation			Lower radiation				
	Mean	SE	*n*	Mean	SE	*n*	Mean	SE	*n*		
	81.25	7.21	8	73.84	3.65	26	86.75	6.65	7	1.51	0.23
Working memory										*F* value	*p* value
Laterality	Unilateral SNHL			Bilateral SNHL							
	Mean	SE	*n*	Mean	SE	*n*					
	79.98	5.00	11	78.64	2.76	36				0.06	0.82
Treatment	Chemotherapy			Higher radiation			Lower radiation				
	Mean	SE	*n*	Mean	SE	*n*	Mean	SE	*n*		
	86.08	5.65	10	74.24	3.04	29	87.17	5.57	8	2.93	0.06
Processing speed										*F* value	*p* value
Laterality	Unilateral SNHL			Bilateral SNHL							
	Mean	SE	*n*	Mean	SE	*n*					
	74.38	4.12		75.37	2.31					0.04	0.84
Treatment	Chemotherapy			Higher radiation			Lower radiation				
	Mean	SE	*n*	Mean	SE	*n*	Mean	SE	*n*		
	81.32	4.58	10	70.56^d^	2.51	28	83.38^d^	4.47	8	4.04	**0.02**

Notes

Results from ANCOVAs, comparing intellectual outcome across patients with SNHL (*n* = 48) stratified by: (1) laterality (unilateral SNHL/bilateral SNHL) and (2) treatment (chemotherapy/higher radiation/lower radiation). Age at diagnosis was included as a covariate; adjusted group means and standard errors (SE) are provided. Due to differences in tests used to assess intellectual outcome, *n*'s vary across groupings. Post hoc pairwise analyses were corrected for multiple comparisons using the modified Hochberg procedure. Matching letters indicate significant post hoc pairwise comparisons among groups from the three group comparison: ^a^ *p* = 0.05, ^b^ *p* = 0.004, ^c^ *p* = 0.02, ^d^ *p* = 0.05. n = sample size.

Bolded values indicate significant *P*‐values (i.e. *P* < 0.05).

### 3.4 Exploratory analyses

#### 3.4.1 Patients treated with chemotherapy alone who develop SNHL may be vulnerable to poor intellectual outcomes, especially working memory

**FIGURE 3:**
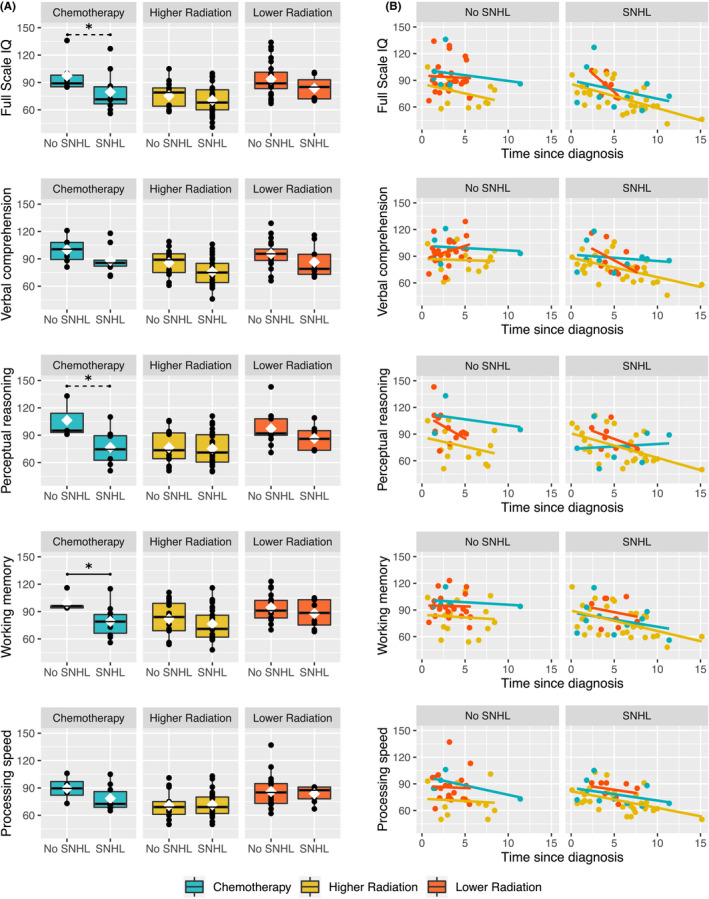
(A) Boxplots showing all data points for measures of intellectual function, for patients in each treatment group (chemotherapy, higher radiation, lower radiation) separated by hearing status (SNHL vs. No SNHL). In the chemotherapy group, prior to correction for multiple comparisons, patients that developed SNHL had lower full scale IQ, perceptual reasoning and working memory scores than patients with intact hearing (all *p *< 0.05). Following correction for multiple comparisons, working memory remained significantly different between the SNHL and No SNHL groups (*p = 0.03*). Refer to Table 4 for full results. Patients treated with higher radiation and lower radiation did not differ in their scores on any measure of intellectual function, regardless of SNHL. Black line, median; white diamond, mean. Significance: dashed line, uncorrected; solid line, corrected. (B) The association between measures of intellectual function and time since diagnosis were plotted for patients in each group; this was done to visualize how much time since diagnosis had elapsed when the assessment points were acquired

Uncorrected Mann–Whitney *U* tests indicated that patients treated with chemotherapy alone who developed SNHL had lower full scale IQ (*p* = 0.03), perceptual reasoning (*p *= 0.03), and working memory (*p *= 0.01) scores than patients with intact hearing (Table 4, Figure 3A). Following correction for multiple comparisons using the modified Hochberg procedure, working memory remained significantly different between the SNHL and no‐SNHL groups (*p = 0.03*). Plots of individual patient test score data in relation to time since diagnosis are provided (Figure 3B); visually it is evident that patients in the SNHL and no‐SNHL groups were assessed at similar time points following diagnosis. We note that intellectual outcome did not differ according to hearing status, for patients in either radiation group (Table 4).

**TABLE 4:** Intellectual outcome by hearing status, in the chemotherapy, higher radiation and lower radiation treatment groups

	No‐SNHL			SNHL					
	*n*	Median (IQR)		*n*	Median (IQR)	*U*	Effect size—*r*	*p* (*uncorrected*)	*p (* *corrected)*
Chemotherapy group (*n* = 16)									
Full scale IQ	6	89.0 (12.8)		10	71.5 (18.75)	47.5	0.48	*0.03*	0.07
Verbal comprehension	6	100.5 (17.8)		10	85.5 (6.50)	44.4	0.4	0.06	0.07
Perceptual reasoning	3	95.0 (21.0)		8	74.5 (27.0)	21.5	0.49	*0.03*	0.07
Working memory	5	96.0 (2.0)		10	79.0 (20.5)	46.0	0.65	*0.01*	**0.03**
Processing speed	4	89.5 (15.0)		10	72.5 (17.25)	31.0	0.39	0.07	0.07
Higher radiation group (*n* = 44)									
Full scale IQ	13	79.0 (20.0)		29	68.0 (22.0)	223.5	0.14	0.17	0.59
Verbal comprehension	15	89.0 (20.5)		29	75.0 (21.0)	296.0	0.29	*0.03*	0.13
Perceptual reasoning	14	73.5 (28.25)		26	71.0 (30.0)	193.0	0.05	0.38	0.61
Working memory	15	84.0 (30.0)		29	71.0 (24.0)	252.5	0.13	0.20	0.59
Processing speed	14	69.0 (14.0)		28	69.0 (18.0)	186.0	−0.04	0.61	0.61
Lower radiation group (*n* = 34)									
Full scale IQ	24	89.0 (18.0)		7	85.0 (21.0)	111.5	0.22	0.10	0.40
Verbal comprehension	24	95.5 (12.5)		9	79.0 (22.0)	149.0	0.29	*0.05*	0.25
Perceptual reasoning	13	92.0 (18.0)		7	86.0 (21.5)	59.5	0.19	0.14	0.40
Working memory	22	91.0 (19.3)		8	88.5 (27.8)	106.5	0.15	0.20	0.40
Processing speed	20	85.0 (21.8)		8	87.5 (13.0)	77.5	−0.02	0.56	0.56

Abbreviations: IQR, interquartile range; SNHL, sensorineural hearing loss.

Results from Mann‐Whitney U tests, comparing intellectual outcome across patients with and without SNHL in the chemotherapy, higher radiation and lower radiation groups separately. Due to differences in tests used to assess intellectual outcome, n's vary across groupings. *p*‐values were corrected using the modified Hochberg procedure, to account for the multiple comparisons made. IQR: interquartile range, SNHL: sensorineural hearing loss. Bold values: significant corrected *p*‐values. Italic values: significant uncorrected *p*‐values.

## 4 DISCUSSION

Hearing plays a vital role in the acquisition of speech and verbal language, and in the achievement of developmental milestones. Growing evidence on the impact of SNHL on speech‐language, social‐emotional, cognitive development, and academic achievement supports the need for early identification, complete assessment, and support of children with SNHL.^22^, ^30^, ^31^ Here, we demonstrate that SNHL is associated with poor intellectual outcome in children treated for PF embryonal brain tumors, particularly when they are treated with high doses of chemotherapy followed by high dose carboplatin—though this last finding must be considered preliminary due to sample size constraints. Children who developed SNHL after treatment with radiation sparing approaches had significantly lower perceptual reasoning and working memory than those who did not develop SNHL. In previous samples, radiation therapy and presence of SNHL were confounded. For example, in one study 39% of children with medulloblastoma had severe SNHL, which was associated with poorer cognitive outcomes. However, their sample included patients treated with and without radiation,^9^ making it difficult to disentangle the contributing role of radiation versus SNHL to poor intellectual outcomes. An advantage of our study is that we evaluated the impact of SNHL specifically in a group of patients who were treated without radiation.

It is crucial to recognize that using a chemotherapy only approach in attempt to avoid radiation therapy is associated with significant ototoxicity that itself increases the risk for severe intellectual deficits. Although survival with this approach appears to be superior to other lower intensity protocols,^32^, ^33^ careful neurocognitive monitoring is warranted in these patients, as well as early hearing support when SNHL develops. We note that the rate of SNHL in our sample (51%) is somewhat higher than noted in some of the existing literature.^13^ Two factors may account for this apparent discrepancy. First, differences in timing of the audiological assessment may be relevant here as we conducted audiological assessments at a later time point (i.e., 5 years post‐treatment) and some of our sample only developed SNHL at a later time point. Second, previous studies have considered samples of embryonal brain tumor patients treated with radiation and chemotherapy, whereas we are including a sample that includes chemotherapy only, and therefore a larger proportion of our sample received higher doses of platinum‐based therapies than earlier studies focused on the incidence of SNHL. Therefore, our sample is unique and is particularly relevant to populations treated with higher doses of platinum‐based therapies. Finally, we noted that the rate of SNHL in our sample aligns with a large multicenter study, conducted in a sample of 1481 patients who received cisplatin.^34^ In this study, the authors demonstrated that 43.8% of all children treated for cancer developed cisplatin‐induced SNHL, and that rates were even higher for brain tumor patients (50.9%), and children treated <5 years (59.4%). Based on our findings, with significantly lower perceptual reasoning and working memory identified in children treated with chemotherapy only who experience severe SNHL, these children are at risk for school problems; low working memory can affect a child's concentration and ability to follow instructions, and low perceptual reasoning can render a child less capable of using effective problem‐solving strategies.

It is well established that on average children born with unilateral SNHL are at risk for poor cognitive performance.^35^ To date, the impact of unilateral versus bilateral SNHL in children treated for embryonal tumors had not been examined. Here, we found that patients with unilateral SNHL had intellectual outcomes that were as poor as patients with bilateral SNHL. Our findings have implications for increased monitoring and hearing support in patients treated for embryonal tumors and suggest that even unilateral SNHL warrants immediate and aggressive rehabilitation upon detection.

We recognize the following limitations to our study. First, different test versions were employed to assess intelligence over time. It is possible that test version is confounded with group. Second, our sample size of children treated with chemotherapy only is small, and our analysis comparing those with and without SNHL may be underpowered as a result. Thus, the interpretability of our findings are limited, and require further validation in a larger cohort. Third, we did not assess post‐surgical complications such as posterior fossa syndrome, degree, and duration of hydrocephalus. Fourth, our study was retrospective in nature and biases may exist in the sample selected. Prospective studies designed to investigate the impact of hearing losing in larger samples of children treated without radiation are required. Finally, the heterogeneity of the population in terms of age, diagnoses, disease extent, and radiation dose reflect the reality of clinical practice, as does the variety of protocols used. During the time period, 12 different protocols were used (Table S1). However, protocols for children with embryonal tumors are relatively consistent with regard to dosing, schedules, and dose adjustments concerning cisplatin and/or carboplatin. Despite these limitations, our study demonstrates an association between severe SNHL and poor intellectual outcome.

## 5 CONCLUSIONS

Our findings highlight the detrimental effect of SNHL on intellectual outcome in children treated for embryonal brain tumors, and suggest early hearing interventions should be prioritized and examined for their potential to improve long‐term outcome, particularly among patients treated with radiation sparing approaches. There is recent evidence that otoprotectants are associated with a lower incidence of cisplatin‐induced hearing loss in children treated with chemotherapy.^36^, ^37^, ^38^ Identification of specific patient populations who may be at risk, by screening for genetic susceptibility to cisplatin‐induced SNHL, could be a useful tool to try and minimize patients at risk for SNHL.^17^, ^39^ However, such screening has not been implemented in clinical practice, reflecting the challenges associated with identification of high risk patients versus the risks of altering treatments with proven efficacy. Lastly, early intervention is crucial to optimize hearing abilities. This may be achieved through amplification with hearing aids or even CI where indicated, as MRI compatible CIs are now available. Additionally, interventions centered around developing alternate modes of communication, such as sign language, and facilitating patient involvement with resources and/or schooling available to children within the broader deaf community should be prioritized. Regardless of modality used, early intervention is both feasible and warranted for this group of patients.^40^ Interventions such as sodium thiosulfate and other putative otoprotective agents should be urgently prioritized for evaluation in prospective clinical trials.^38^

As long‐term survival continues to improve in children treated for embryonal brain tumors, we are increasingly faced with the long‐term consequences of its treatments. In an effort to help these children not only survive longer but to thrive, we need to begin with an understanding of the treatment‐related variables that negatively impact their development. This study does just that by emphasizing the importance of SNHL on intellectual outcome in children treated for embryonal brain tumors. Efforts should be made to integrate hearing sparing strategies in future protocols for this vulnerable population. This includes the use of otoprotectants that have demonstrated a significant benefit in clinical trials. It should also include a careful review of the regimens used and a search for alternative regimens without platinum compounds.

## ETHICAL APPROVAL STATEMENT

This study was approved by the Research Ethics Board at The Hospital for Sick Children prior to commencement and conforms to the Declaration of Helsinki and the Code of Ethics of the Canadian Psychological Association.

## CONFLICT OF INTEREST

None.

## Supporting information

Table S1

Table S2

Table S3

## Data Availability

In accordance with terms of consent given by participants or legal guardians, raw data utilized in this study are not available for sharing. Even so, aggregate data (mean values) are available upon request. This policy is in accordance with the Research Ethics Board at The Hospital for Sick Children.
