# Supplementary material for: Hearing loss and intellectual outcome in children treated for embryonal brain tumors: Implications for young children treated with radiation sparing approaches
Source: Cancer Med. 2021 Sep 4;10(20):7111–25. doi: 10.1002/cam4.4245 (PMC8525144; doi:10.1002/cam4.4245)
Supplement: Supplementary file 1 — Table S1 [file CAM4-10-7111-s001.docx]

| **Protocol** | **Age (years)** | **Diagnosis** | **Therapy** | **Reference** |
| --- | --- | --- | --- | --- |
| **ACNS1221** | < 4 | Medulloblastoma | Carboplatin 1995-3000 mg/m^2^ | Not published, clinicaltrialc.gov; NCT02017964 |
| **ACNS0332** | ≥ 3 and < 22 | HR Medulloblastoma | CSI 36 Gy, primary site 55.8 Gy  Carboplatin 1050 mg/ m^2^ (by randomization)  Cisplatin 450 mg/m^2^ | Not published, clinicaltrials.gov; NCT00392327 |
| **SJMB03** | ≥ 3 and < 22 | AR and HR Medulloblastoma  /PNET/ATRT | CSI 23.4 Gy or 36-39.6 Gy, primary site 55.8 Gy  Cisplatin 300 mg/m^2^ | Not published, clinicaltrials.gov;  NCT00085202 |
| **SJMB96** | ≥ 3 and < 22 | HR and AR Medulloblastoma | CSI 23.4 Gy or 36-39.6 Gy, primary site 55.8 Gy  Cisplatin 300 mg/m^2^ | Gajjar et al., 2006 |
| **CCG 9631** | ≥ 3 and < 22 | HR Medulloblastoma | CSI 36-39.6 Gy, primary site 55.8 Gy  Cisplatin 270 mg/m^2^ | Esbenshade et al., 2017 |
| **CCG9961** | ≥ 3 | AR Medulloblastoma | CSI 23.4 Gy and 55.8 Gy to primary site  Cisplatin 600 mg/m^2^ | Packer et al., 2006 |
| **99703** | ≥ 0.5 and < 3 | Malignant brain tumors | Cisplatin 10.5 mg/kg  Carboplatin 102 mg/kg | Cohen et al., 2015 |
| **MOPP** | ≥ 1 and < 22 | Medulloblastoma | CSI 25-30 Gy and primary site 54-54.4 Gy | Krischer et al., 1991 |
| **Baby POG** | < 3 | Malignant brain tumors | Cisplatin 8 mg/kg  CSI 24-35.2 and 50-54 to primary site | Duffner et al., 1999 & Duffner et al., 1993 |
| **Head Start II** | < 10 | Malignant brain tumors | Cisplatin 17.5 mg/kg 525 mg/m^2^  Carbopaltin 1000 mg/m2 | Chi et al., 2004 |
| **ACNS0334** | < 3 | Supratentorial PNET/HR Medulloblastoma | Cisplatin 21 mg/kg  Carboplatin 102 mg/kg | Not published, clinicaltrials.gov; NCT00336024 |
| **ACNS0331** | ≥ 3 and < 22 | AR Medulloblastoma | CSI 18 vs 23.4 Gy. 54 Gy to primary site  Cispaltin 450 mg/m^2^ | Not published, clinicaltrials.gov; NCT00085735 |

AR = average risk; ATRT = atypical teratoid rhabdoid tumor; CSI = craniospinal irradiation; Gy = gray; HR = high risk; PNET = primitive neuroectodermal tumor
