# Supplementary material for: Hearing loss and intellectual outcome in children treated for embryonal brain tumors: Implications for young children treated with radiation sparing approaches
Source: Cancer Med. 2021 Sep 4;10(20):7111–25. doi: 10.1002/cam4.4245 (PMC8525144; doi:10.1002/cam4.4245)
Supplement: Supplementary file 2 — Table S2 [file CAM4-10-7111-s002.docx]

A. Tests and versions administered the three treatment groups

|  | **Chemotherapy** | **Higher Radiation** | **Lower Radiation** |
| --- | --- | --- | --- |
|  | **n = 16** | **n = 44** | **n = 34** |
| WAIS-IV | 2 (12.5) | 7 (15.9) | 0 (0.0) |
| WASI | 0 (0.0) | 0 (0.0) | 1 (2.9) |
| WISC-III | 0 (0.0) | 5 (11.4) | 0 (0.0) |
| WISC-IV | 5 (31.2) | 28 (63.6) | 14 (41.2) |
| WISC-IV/WASI | 0 (0.0) | 0 (0.0) | 5 (14.7) |
| WISC-V | 1 (6.2) | 0 (0.0) | 0 (0.0) |
| WJ-III | 0 (0.0) | 1 (2.3) | 9 (26.5) |
| WPPSI-III | 1 (6.2) | 1 (2.3) | 2 (5.9) |
| WPPSI-IV | 7 (43.8) | 1 (2.3) | 3 (8.8) |
| SBIS | 0 (0.0) | 1 (2.3) | 0 (0.0) |

Values refer to n (%)

B. Tests and versions administered in the SNHL/No SNHL subgroups

|  | **Chemotherapy** | | **Higher Radiation** | | **Lower Radiation** | |
| --- | --- | --- | --- | --- | --- | --- |
|  | **No SNHL** | **SNHL** | **No SNHL** | **SNHL** | **No SNHL** | **SNHL** |
|  | **n = 6** | **n = 10** | **n = 15** | **n = 29** | **n = 25** | **n = 9** |
| WAIS-IV | 0 (0.0) | 2 (20.0) | 2 (13.3) | 5 (17.2) | 0 (0.0) | 0 (0.0) |
| WASI | 0 (0.0) | 0 (0.0) | 0 (0.0) | 0 (0.0) | 1 (4.0) | 0 (0.0) |
| WISC-III | 0 (0.0) | 0 (0.0) | 5 (33.3) | 0 (0.0) | 0 (0.0) | 0 (0.0) |
| WISC-IV | 1 (16.7) | 4 (40.0) | 7 (46.7) | 21 (72.4) | 9 (36.0) | 5 (55.6) |
| WISC-IV/WASI | 0 (0.0) | 0 (0.0) | 0 (0.0) | 0 (0.0) | 5 (20.0) | 0 (0.0) |
| WISC-V | 0 (0.0) | 1 (10.0) | 0 (0.0) | 0 (0.0) | 0 (0.0) | 0 (0.0) |
| WJ-III | 0 (0.0) | 0 (0.0) | 0 (0.0) | 1 (3.4) | 7 (28.0) | 2 (22.2) |
| WPPSI-III | 1 (16.7) | 0 (0.0) | 1 (6.7) | 0 (0.0) | 0 (0.0) | 2 (22.2) |
| WPPSI-IV | 4 (66.7) | 3 (30.0) | 0 (0.0) | 1 (3.4) | 3 (12.0) | 0 (0.0) |
| SBIS | 0 (0.0) | 0 (0.0) | 0 (0.0) | 1 (3.4) | 0 (0.0) | 0 (0.0) |

Values refer to n (%)
