# Supplementary material for: Hearing loss and intellectual outcome in children treated for embryonal brain tumors: Implications for young children treated with radiation sparing approaches
Source: Cancer Med. 2021 Sep 4;10(20):7111–25. doi: 10.1002/cam4.4245 (PMC8525144; doi:10.1002/cam4.4245)
Supplement: Supplementary file 3 — Table S3 [file CAM4-10-7111-s003.docx]

|  | **Chemotherapy**  n = 10 | | **Higher Radiation**  n = 29 | | **Lower Radiation**  n = 9 | |  |
| --- | --- | --- | --- | --- | --- | --- | --- |
|  |  |  |  |  |  |  | P value |
|  | n | (%) | n | (%) | n | (%) |  |
| **Sex** |  |  |  |  |  |  | 0.52 |
| Male | 8 | (80.0) | 20 | (69.0) | 5 | (55.6) |  |
| Female | 2 | (20.0) | 9 | (31.0) | 4 | (44.4) |  |
| **Tumor Type** |  |  |  |  |  |  | **0.03** |
| Medulloblastoma | 5 | (50.0) | 25 | (86.2) | 7 | (77.8) |  |
| ATRT | 5 | (50.0) | 1 | (3.4) | 1 | (11.1) |  |
| Pineoblastoma | 0 | (0.0) | 2 | (6.9) | 1 | (11.1) |  |
| PNET | 0 | (0.0) | 1 | (3.4) | 0 | (0.0) |  |
| **Hydrocephalus** |  |  |  |  |  |  | 0.94 |
| Yes | 9 | (90.0) | 25 | (86.2) | 8 | (88.9) |  |
| No | 1 | (10.0) | 4 | (13.8) | 1 | (11.1) |  |
| **Shunt** |  |  |  |  |  |  | 0.25 |
| Yes | 6 | (60.0) | 12 | (41.4) | 2 | (22.2) |  |
| No | 4 | (40.0) | 17 | (58.6) | 7 | (77.8) |  |
| **SNHL** |  |  |  |  |  |  | NA |
| Chang grade < 2b | 0 | (0.0) | 0 | (0.0) | 0 | (0.0) |  |
| Chang grade ≥ 2b | 10 | (100.0) | 29 | (100.0) | 9 | (100.0) |  |
| **Hearing aid** |  |  |  |  |  |  | 0.08 |
| Yes | 8 | (80.0) | 12 | (41.4) | 6 | (66.7) |  |
| No | 2 | (20.0) | 17 | (58.6) | 3 | (33.3) |  |
|  | Mean | (SD) | Mean | (SD) | Mean | (SD) |  |
| **Chemotherapy** |  |  |  |  |  |  |  |
| ∑CDDP, mg/m^2^ | 259.57 | (120.43) | 328.08 | (112.90) | 297.50 | (63.75) | 0.22 |
| ∑Carboplatin g/m^2^ | 1939.50^a,b^ | (1138.82) | 88.97^a^ | (338.79) | 566.67^b^ | (1153.00) | **< 0.001** |
| **Age at Testing, years** | 7.56^c^ | (3.28) | 13.15^c^ | (3.52) | 10.40 | (3.50) | **< 0.001** |
| **Age at Diagnosis, years** | 2.57^d^ | (1.33) | 7.53^d^ | (3.78) | 6.10 | (3.60) | **< 0.001** |
| **Time Since Diagnosis, years** | 4.99 | (3.58) | 5.62 | (3.64) | 4.80 | (1.59) | 0.76 |
| **Time Since SNHL, years** | 3.57 | (2.89) | 2.84 | (2.78) | 3.31 | (1.27) | 0.72 |

ATRT = atypical teratoid rhabdoid tumor; CDDP = cisplatin; PNET = primitive neuroectodermal tumor; SNHL = sensorineural hearing loss (Chang grade < 2b = No SNHL; Chang grade ≥ 2b = SNHL); SD = standard deviation. Matching letters indicate groups that differed in the three group comparisons: ^a-d^P < 0.001.
